# Simultaneous Determination of As, Bi, Sb, Se, Te, Hg, Pb and Sn by Small-Sized Electrothermal Vaporization Capacitively Coupled Plasma Microtorch Optical Emission Spectrometry Using Direct Liquid Microsampling

**DOI:** 10.3390/molecules26092642

**Published:** 2021-04-30

**Authors:** Simion Bogdan Angyus, Erika Levei, Dorin Petreus, Radu Etz, Eniko Covaci, Oana Teodora Moldovan, Michaela Ponta, Eugen Darvasi, Tiberiu Frentiu

**Affiliations:** 1 Faculty of Chemistry and Chemical Engineering, Babes-Bolyai University, Arany Janos 11, 400028 Cluj-Napoca, Romania; bogdan.angyus@gmail.com (S.B.A.); eniko.covaci@ubbcluj.ro (E.C.); michaela.ponta@ubbcluj.ro (M.P.); darvasi.jeno@gmail.com (E.D.); 2 Research Center for Advanced Chemical Analysis, Instrumentation and Chemometrics–Analytica, Babes-Bolyai University, Arany Janos 11, 400028 Cluj-Napoca, Romania; 3 National Institute for Research and Development of Optoelectronics INOE 2000 INCD Bucharest, Research Institute for Analytical Instrumentation, Donath 67, 400293 Cluj-Napoca, Romania; erika.levei@icia.ro; 4 Faculty of Electronics, Telecommunications and Information Technology, Technical University of Cluj-Napoca, George Baritiu 26–28, 40002 Cluj-Napoca, Romania; dorin.petreus@ael.utcluj.ro (D.P.); radu.etz@ael.utcluj.ro (R.E.); 5 Department Cluj-Napoca, Emil Racovita Institute of Speleology, Clinicilor 5, 4000006 Cluj-Napoca, Romania; oanamol35@gmail.com; 6 Romanian Institute of Science and Technology, Saturn 24–26, 400504 Cluj-Napoca, Romania

**Keywords:** capacitively coupled plasma, optical emission spectrometry, electrothermal vaporization, direct liquid microsampling, cave sediment, river sediment, water sediment

## Abstract

The simultaneous determination of chemical vapor-generating elements involving derivatization is difficult even by inductively coupled plasma optical emission spectrometry or mass spectrometry. This study proposes a new direct liquid microsampling method for the simultaneous determination of As, Bi, Se, Te, Hg, Pb, and Sn, using a fully miniaturized set-up based on electrothermal vaporization capacitively coupled plasma microtorch optical emission spectrometry. The method is cost-effective, free from non-spectral interference, and easy to run by avoiding derivatization. The method involves the vaporization of analytes from the 10 µL sample and recording of episodic spectra generated in low-power (15 W) and low-Ar consumption (150 mL min^−1^) plasma microtorch interfaced with low-resolution microspectrometers. Selective vaporization at 1300 °C ensured the avoidance of non-spectral effects and allowed the use of external calibration. Several spectral lines for each element even in the range 180–210 nm could be selected. Generally, this spectral range is examined with large-scale instrumentation. Even in the absence of derivatization, the obtained detection limits were low (0.02–0.75 mg kg^−1^) and allowed analysis of environmental samples, such as cave and river sediments. The recovery was in the range of 86–116%, and the accuracy was better than 10%. The method is of general interest and could be implemented on any miniaturized or classical laboratory spectrometric instrumentation.

## 1. Introduction

The increasing interest in the determination of As, Bi, Sb, Se, Te, Hg, Pb, and Sn is closely related to their special uses in emerging technologies for the synthesis of materials or medicine [1,2,3,4]. On the other hand, their involvement in various enzyme-powered pathways should be considered. The crucial role of Se in human, animal, and plant health [5,6] as well as the high toxicity of Pb, Hg, As, and Sb are well known [7,8,9,10]. The quantification of these elements in environmental and biological samples, food, and materials by spectrometric methods is not an easy task given the low sensitivity of the spectral lines, spectral and non-spectral interferences even in the case of high-performance techniques such as graphite or quartz furnace atomic absorption spectrometry (GFAAS, QFAAS), atomic fluorescence spectrometry (AFS), and inductively coupled plasma optical emission or mass spectrometry (ICP OES, ICP-MS). Several sample preparation protocols have been developed and investigated in order to overcome these impediments. Undoubtedly, the classical hydride generation (HG)/cold vapor (CV) generation using NaBH_4_ and SnCl_2_ [11,12,13] combined with spectrometric methods are the most common approaches. Such examples are HG-AFS and CV-AFS [14,15,16,17,18,19], HG coupled with high-resolution continuum source electrothermal atomic absorption spectrometry or quartz furnace atomic absorption spectrometry (HG-HR-CS-ETAAS, HG-HR-CS-QFAAS) [20,21,22] and graphite furnace atomic absorption spectrometry (HG-GFAAS) [23]. Other ways are HG-ICP OES [24], HG-ICP-MS [25,26], and hydride generation laser-induced breakdown spectrometry (HG-LIBS) [27]. In addition, the use of UV photochemical vapor generation (UV-PVG) [28,29] coupled with spectrometric methods such as UV-PVG-AFS [30], UV-PVG-GFAAS [31,32], and UV-PVG-ICP-MS [33,34] resulted in enhanced sensitivity and lower susceptibility to non-spectral interference compared to conventional HG. Recently, hydride generation liquid anode/cathode atmospheric pressure glow discharge optical emission spectrometry (HG-FLA-APGD-OES, HG-FLC-APGD-OES) in a unique set-up was investigated by Pohl et al. in Poland [35,36]. The simultaneous determination of chemical vapor generating elements is still a challenge and a highly desirable goal; however, it is difficult to achieve because of the different conditions for prereduction and derivatization. In the case of these methods, a significant number of operating parameters should be optimized in order to obtain the best figures of merit. Typically, single-element or fit-for-purpose procedures are available. Atomic absorption spectrometry offers only sequential analysis. Instead, ICP OES and ICP-MS can perform simultaneous determination, provided that the strategy considers the grouping of elements according to prereduction and derivatization conditions or a compromise solution, which may affect the performance for some elements [11,12,13,24,35]. The direct determination of chemical vapor-generating elements by avoiding derivatization was also attempted. Therefore, mercury determination by thermal decomposition atomic absorption spectrometry (TD-AAS) [37,38], liquid/solid sampling with electrothermal vaporization (ETV) in GFAAS [39,40,41], or the emergent HR-CS-ETAAS using Xe lamp [42,43] were successfully applied. At the same time, ICP OES [44] and ICP-MS [45,46] using pneumatic and ultrasonic nebulization, or solid sampling by laser ablation (LA-ICP-MS) [47] and LIBS OES [48] provide simultaneous direct determination without derivatization. The concept of ETV miniaturized systems using a platform or metal coil for sample introduction in liquid or solid form was successfully implemented for the quantification of elements generating chemical vapor without derivatization by ETV-AFS [49], ETV-ICP OES, and ETV-ICP-MS [50,51,52,53,54]. The instrumentation associated with ICP OES and ICP-MS provides excellent sensitivity; however, it is conceived as laboratory-scale equipment that is quite expensive in terms of energy and argon support gas. Alternatively, the technology of fully miniaturized spectrometric instruments incorporating microplasma has developed considerably and has become very attractive for specific analytical applications. The benefits are related to the unique characteristics of the devices and advantages in terms of portability, low operation power, low Ar or He consumption, and analytical performance close to classical laboratory instrumentation [55,56,57,58]. However, because of the low operating power, microplasmas exhibit low tolerance for water that affects the stability of the discharge and excitation capability. Consequently, the introduction of liquid samples is still problematic unless plasma is generated at the surface of the sample as one of the electrodes, either the cathode or anode [58]. Karanassios’ group developed miniature ETV devices with metallic filaments that were further considered by other researches as a universal and easy approach for the introduction of liquid sample in microplasmas, thus widening the applicability of these sources [59,60,61,62]. In the same direction, our group reported the development of a fully miniaturized analytical system consisting of a small-sized Rh filament electrothermal vaporization device interfaced with a low power (15 W) and low argon consumption (150 mL min^−1^) capacitively coupled plasma microtorch for detection by optical emission spectrometry using a low-resolution microspectrometer (SSETV-µCCP-OES). Its usefulness was demonstrated for the simultaneous determination of several toxic elements in liquid microsamples (environment and food) as an alternative to ICP OES with pneumatic nebulization and GFAAS [63,64,65,66].

The SSETV-µCCP tandem incorporating the Rh-coiled filament was easy to interface due to similar demands in terms of operation power (11 W for filament heating, 15 W for plasma source) and Ar flow for the two components (100–150 mL min^−1^). The tandem has been found ideal for the introduction of liquid microsamples into the microplasma torch due to the instantaneous heating of the Rh filament, which yields an efficient vaporization of the microsample and high flow of analytes into plasma. This allowed a simultaneous determination of elements of concern for the environment and food, including harmful elements of high priority (Cd, Pb, Hg), with very good detection limits. There are several benefits of using Rh for the filament, as this material is easily workable, and it exhibits high resistance to oxygen and aqua regia used for sample mineralization. Thus, it is not necessary to create a hydrogen-protecting atmosphere, which causes plasma destabilization and an increase of the background spectrum of the Ar plasma in the UV range with a detrimental effect on the detection limits. Unfortunately, the SSETV-µCCP-OES method was prone to non-spectral interferences arising from the mineral matrix when the Rh filament was heated to 1500 °C to accomplish vaporization of less volatile analytes. To avoid the matrix interference, quantification was performed using the standard addition. However, heating the filament to a lower temperature (1300 °C) provided a selective vaporization of Hg and thus, a new opportunity for the determination of total Hg and CH_3_ Hg^+^ by SSETV-µCCP-OES using external calibration [67]. New validated methods, free from non-spectral interference using miniaturized instrumentation incorporating plasma sources continue to be a challenge and a necessity. Considering these working hypotheses, this study aimed to develop a new method for the simultaneous determination of As, Bi, Sb, Se, Te, Hg, Pb, and Sn in environmental samples by direct liquid microsampling using the SSETV-µCCP-OES prototype instrument. The spectral and analytical evaluation of the new set-up was performed by examining the plasma microtorch with two low-resolution microspectrometers. The SSETV-µCCP-OES tandem was optimized in terms of drying and vaporization temperatures for a selective, but effective vaporization, plasma operating power, Ar flow, and observation height. The processing of the transient signals and background correction as well as method validation in terms of detection limit (LOD), precision, and accuracy are presented. The absence of non-spectral interference was checked through analyte recovery in certified reference materials using external calibration and standard addition. The capability and utility of the innovative SSETV-µCCP-OES system was demonstrated by analyzing cave and river sediment samples. The results have significance for analytical practice, as it emphasizes the opportunity of using cost-effective miniaturized laboratory instrumentation for the determination of As, Bi, Sb, Se, Te, Hg, Pb, and Sn, and it is of interest for the environment, without derivatization, as an alternative to classical laboratory analytical systems. In addition, this approach is a topic of relevance and general interest and could be implemented on any miniaturized or classical spectrometric instrumentation.

## 2. Results

### 2.1. Characteristics of the Emission Spectrum of Elements

**Figure 1:**
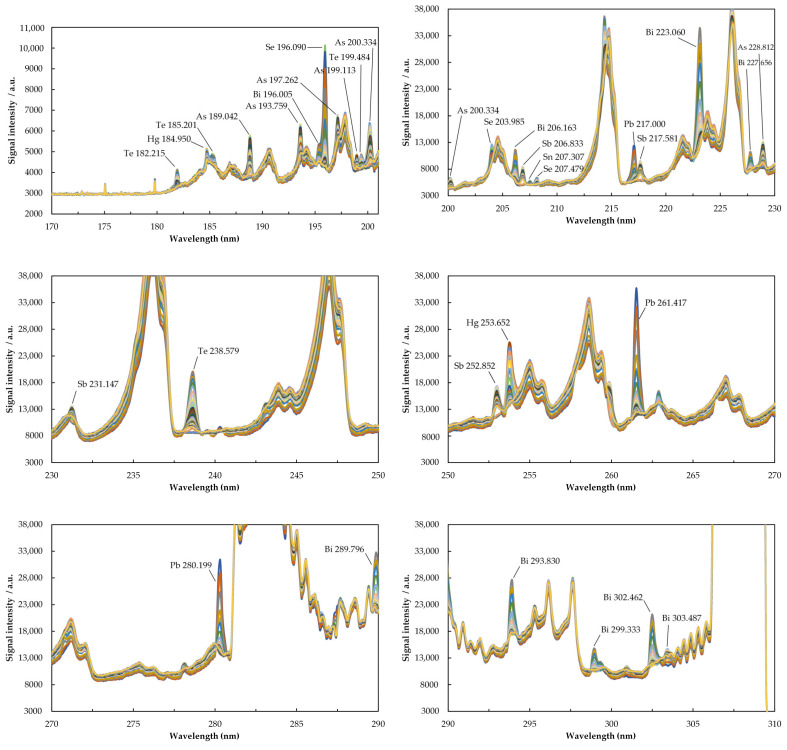
Episodic emission spectra of As, Bi, Sb, Se, Te, Hg, Pb, and Sn in SSETV-µCCP-OES recorded with the Ocean Optics Maya2000 Pro microspectrometer in High Speed Acquisition mode. Conditions: 10 µL sample volume containing 3 µg mL^−1^ As, Bi, Se, Te, and Sn; 1 µg mL^−1^ Pb and Sb; and 0.2 µg mL^−1^ Hg; drying temperature: 80 °C for 180 s; electrothermal vaporization temperature: 1300 °C for 10 s; integration time per episode: 200 ms; plasma power: 15 W; Ar flow rate: 150 mL min^−1^; observation height: 0.8 mm above the electrode tip.

**Figure 2:**
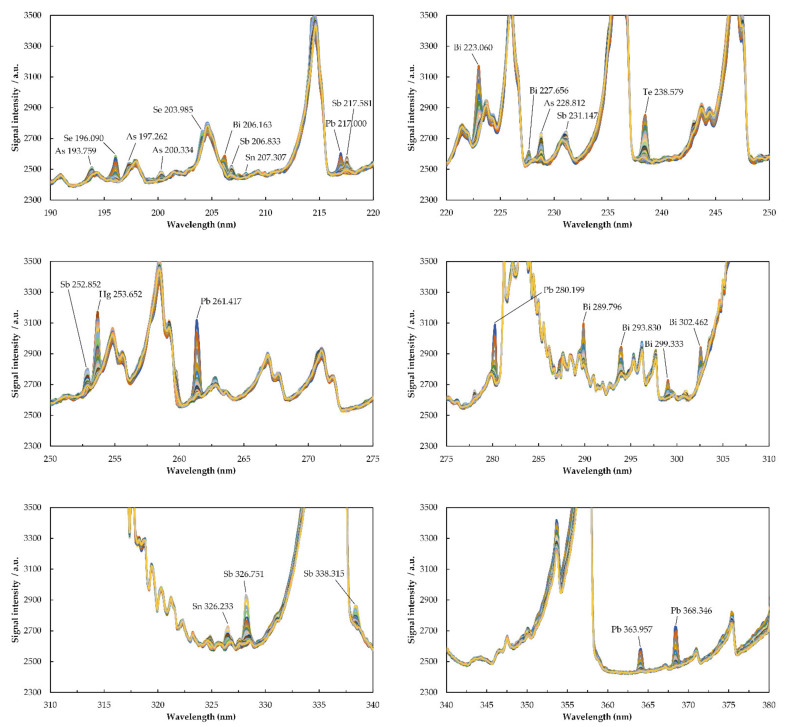
Episodic emission spectra of As, Bi, Sb, Se, Te, Hg, Pb, and Sn in SSETV-µCCP-OES recorded with the Ocean Optics QE65 Pro microspectrometer in High-Speed Acquisition mode. Conditions: 10 µL sample volume containing 3 µg mL^−1^ As, Bi, Se, Te, and Sn; 1 µg mL^−1^ Pb and Sb; and 0.2 µg mL^−1^ Hg; drying temperature: 80 °C for 180 s; electrothermal vaporization temperature: 1300 °C for 10 s; integration time per episode: 200 ms; plasma power: 15 W; Ar flow rate: 150 mL min^−1^; observation height: 0.8 mm above the electrode tip.

Figure 1 and Figure 2 provide the episodic emission spectra recorded simultaneously with Maya2000 Pro and QE65 Pro microspectrometers (200 ms integration time per episode).

**Figure 3:**
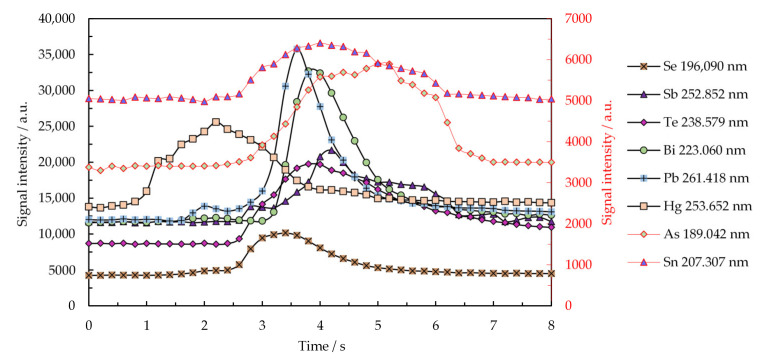
Transient emission signals for the most sensitive lines of As, Bi, Sb, Se, Te, Hg, Pb, and Sn recorded with the Maya2000 Pro microspectrometer. Conditions: 10 µL sample volume containing 3 µg mL^−1^ As, Bi, Se, Te, and Sn; 1 µg mL^−1^ Pb and Sb; and 0.2 µg mL^−1^ Hg; drying temperature: 80 °C for 180 s; electrothermal vaporization temperature: 1300 °C for 10 s; integration time per episode: 200 ms; plasma power: 15 W; Ar flow rate: 150 mL min^−1^; observation height: 0.8 mm above the electrode tip.

An example of a 3D episodic emission spectrum for the most sensitive lines recorded with the Maya2000 Pro microspectrometer is presented in the Supplementary Materials (Figure S1). The excitation energies (eV) of the spectral lines and the relative intensities provided by the two microspectrometers are given in Table 1. The relative intensities corresponding to Maya2000 Pro were calculated by reference to the most sensitive line of each element, while those for the QE65 Pro microspectrometer were given by comparison with Maya2000 Pro. The transient emission signals for the most sensitive lines recorded with the Maya2000 Pro microspectrometer under the optimal working conditions are illustrated in Figure 3.

### 2.2. Optimization of Working Parameters for the SSETV-µCCP-OES Instrument

**Figure 4:**
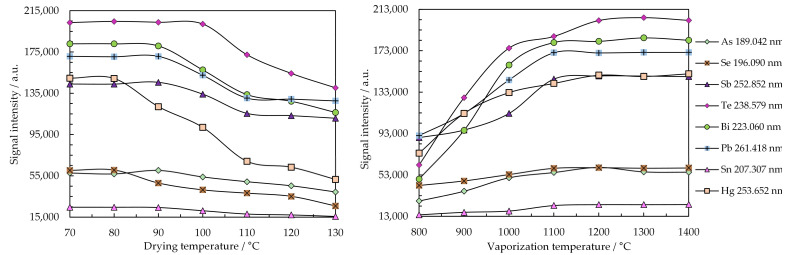
Influence of the drying (**a**) and vaporization (**b**) temperatures on the emission of the most sensitive lines of As, Bi, Sb, Se, Te, Hg, Pb, and Sn registered with the Maya2000 Pro microspectrometer. Conditions: plasma power: 15 W; Ar flow rate: 150 mL min^−1^; observation height: 0.8 mm above the electrode tip; sample: 10 µL containing 3 µg mL^−1^ As, Bi, Se, Te, and Sn; 1 µg mL^−1^ Pb and Sb; and 0.2 µg mL^−1^ Hg.

**Figure 5:**
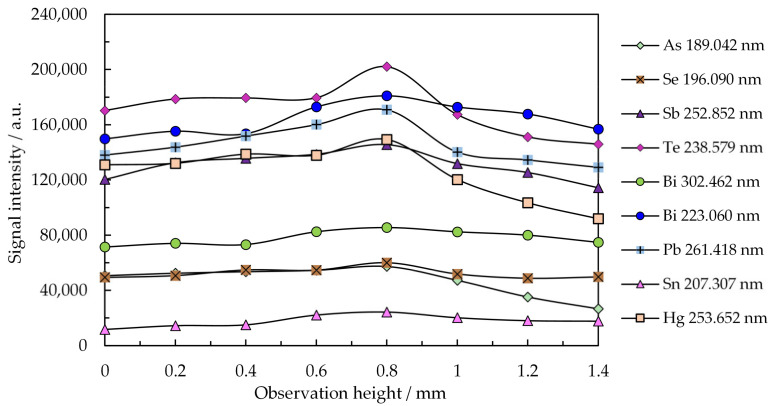
Influence of the observation height in plasma on the emission of the most sensitive lines of As, Bi, Sb, Se, Te, Hg, Pb, and Sn registered with the Maya2000 Pro microspectrometer. Conditions: plasma power: 15 W; Ar flow rate: 150 mL min^−1^; observation height: 0.8 mm above the electrode tip; sample:10 µL containing 3 µg mL^−1^ As, Bi, Se, Te, and Sn; 1 µg mL^−1^ Pb and Sb; and 0.2 µg mL^−1^ Hg.

Figure 4 illustrates the optimization of the drying and vaporization temperatures for a volume of 10 µL microsample. Emission signals were measured with the Maya2000 Pro microspectrometer. Figure 5 refers to the optimization of the observation height for the most sensitive emission lines in plasma operated at 15 W and 150 mL min^−1^ Ar flow rate.

### 2.3. Figures of Merit and Validation of the SSETV-µCCP-OES Method 

The SSETV-µCCP-OES method was characterized in terms of analytical performance, namely, LODs, parameters of the calibration curves established by the net peak area of transient signals and their maximum height (Table 2 and Table 3), accuracy by analyzing several CRMs (Table 4 and Table 5), non-spectral effects of the multimineral matrix and precision in measuring test samples of cave and river sediment. The limits of detection in liquid were calculated using the standard deviation of residuals (s_y/x_) and calibration slope (m) [67], while in the solid based on the sample preparation procedure. The accuracy was estimated by comparing the certified concentrations with found values, for the 95% confidence level. In the case of not certified concentrations or when only one CRM was available for a particular element, the accuracy was established using spike recovery experiments. The non-spectral effects were evaluated by comparing the recoveries found using the standard addition method and external calibration. The composition of the multimineral matrix is provided in the Supplementary Materials (Tables S1 and S2). Precision was expressed as percentage standard deviation (%RSD) from real sample measurements (*n* = 5 parallel measurements).

### 2.4. Application of the SSETV-µCCP-OES Method for the Analysis of Cave and River Sediment

Table 6 shows the results in the analysis of several cave and river sediment test samples obtained by the SSETV-µCCP-OES method using external calibration. 

## 3. Discussions

### 3.1. Excitation Capability of the Capacitively Coupled Plasma Microtorch

The UV background spectrum emitted by the low power and low Ar consumption µCCP consists of molecular emission from NO (A^2^Σ^+^→X^2^Π; E_ex_ = 5.45 eV; 205.28 nm (2,2); 215.49 (9,1); 226.94 nm (0,0); 237.02 nm (1,0); 247.87 nm (2,0); 250.60 nm (5,0)) and OH (A^2^Σ^+^→X^2^Π; E_ex_ = 4.05 eV; 269.11 nm (1,3); 282.90 (0,1); 289.27 nm (1,2); 296.24 nm (2,3); and 308.90 nm (0,0)). In a recent study aiming to determine total Hg and CH_3_ Hg^+^ in food and river sediment by SSETV-µCCP-OES, it was remarked the capability of microplasma to provide several emission lines of Fe, S, and P in the range 180–200 nm [67]. In another study on the simultaneous determination of Ag, As, Cd, Cu, Hg, Pb, Sn, and Zn in soil, it was achieved a selective vaporization of Hg and Ag at 1200–1300 °C, while for the less volatile elements such as Cu and Zn, it was necessary a vaporization temperature of at least 1500 °C [66]. Based on these considerations, the present study tries to answer the question of whether the SSETV-µCCP-OES could be useful for the simultaneous determination of the common chemical vapor-generating elements by selective vaporization from liquid microsamples without derivatization. Previously, the same approach was found convenient for the determination of total mercury and CH_3_ Hg^+^ [67]. The answer is provided in the emission spectra of elements recorded with two low resolution microspectrometers, Maya2000 Pro and QE65 Pro (Figure 1 and Figure 2), and the characteristics of the identified lines (Table 1). It can be noted that the plasma microtorch operated at 15 W is capable of exciting the emission resonance lines of As, Sb, and Pb and non-resonance lines of Bi, Hg, Se, Te, and Sn with excitation energies in the range of 4.33–7.54 eV. The excitation capability of the plasma source for the elements under study was similar to that observed for other elements, when the atomic resonance lines with low excitation energy (<7 eV) were also the most prominent [63,66]. The transitions corresponding to resonance and non-resonance lines with low excitation energy occur through electron–atom collisions and are consistent with the low electron density in the low-power Ar plasma [68]. The emission spectrum poor in lines does not entail analytical limitations, but renders possible the use of low resolution microspectrometers (0.35–0.4 nm FWHM). Thus, the spectrum contains well-defined, intense lines for an element, making possible a versatile selection of the analytical line or even the simultaneous determination of an element at several lines. Compared to previous studies, additional information was obtained for expanding the applicability of the plasma microtorch to the direct determination of chemical vapor-generating elements without derivatization. Moreover, the use of the Maya2000 Pro microspectrometer resulted in an enhancement of sensitivity up to 20–30 times compared to that obtained for the QE65 Pro microspectrometer.

As shown in the 3D spectra (emission intensity–wavelength–time) in the Supplementary Materials (Figure S1), a decrease in the molecular emission of NO and OH occurred during the introduction of the vaporized microsample into the plasma, but there was no observed decrease in the excitation capability of the analytes. The plasma power was consumed to accomplish the atomization and excitation of the analyte atoms. The transient emission (Figure 3) put into evidence a selective vaporization of elements so that the emission signal appeared in a variable number of episodes and reached the maximum in distinct episodes from element to element. The maximum emission of Hg appeared only 2 s after turning on the heating of the Rh filament, while that of As appeared after 5 s. The order of occurrence of the maximum emission in (s) was Hg(2) < Se(3.5) < Pb, Te, Bi, Sb(3.5–4) < As(5). These differences in reaching the maximum signal due to the selective vaporization facilitated the control of non-spectral interferences and avoidance of certain spectral interferences, such as that of Bi 196.005 nm on Se 196.090 nm. Instead, the intense As 228.812 nm line and Cd 228.802 nm could not be resolved by the low-resolution microspectrometer. It could be of interest to further investigate the possibility of eliminating this interference by using a matrix modifier as in the GFAAS method. On the other hand, by accessing the spectral range below 210 nm of low continuous background, which is essential for a high sensitivity, three quite intense resonance lines of As were available for analysis, namely, 189.042 nm, 193.759 nm, and 200.334 nm. The non-resonance lines of Te 182.215 nm, Se 196.090 nm, Bi 206.163 nm, Sb 206.833 nm, and Sn 207.307 nm, free of spectral interference, were also available for analysis. Thus, there is a real opportunity to use fully miniaturized instrumentation encompassing low-resolution microspectrometers and plasma microtorch for the determination of As, Se, Sb, Bi, Sn, and Te based on emission measurements in the range 180–210 nm. This spectral domain is generally accessible only to lab-scale instrumentation, which incorporates quite expensive high-resolution spectrometers.

### 3.2. Capability for Simultaneous Determination by SSETV-µCCP-OES and Influence of Working Conditions

Figure 4a shows the importance of a rigorous control of the drying temperature to avoid loss of the highly volatile analytes. Thus, it was more convenient to conduct microsample drying at 80 °C for a longer period (180 s) than for 80 s at 100 °C. Sample vaporization at 1300 °C for 10 s was also suitable to achieve the selective vaporization of As, Bi, Sb, Se, Te, Hg, Pb, and Sn, as shown in Figure 4b. The vaporization of certain elements, such as Hg, As, Sn, and Se started around 900–1000 °C, and the emission signals appeared in a higher number of episodes than in the case of Pb, Bi, and Te. This behavior is also observable in Figure 3, which illustrates the transient emission signals of the most sensitive lines.

The optimization related to the observation height in plasma operated at 15 W and 150 mL min^−1^ Ar indicated the possibility of the simultaneous determination of As, Bi, Sb, Hg, Se, Te, Pb, and Sn by visualizing the emission at 0.8 mm above the microelectrode tip (Figure 5). The observation height does not critically influence the magnitude of the emission signal, since the excitation energies of the resonance and non-resonance lines of these elements have close values. The low-power Ar discharge is short, and the density of electrons and their energy rapidly decrease over 0–1 mm height above the electrode tip [68]. The maximum emission of analytes observed near the Mo electrode tip indicated that the 7.1 (1st) and 15.6 (2st) eV electrons emitted by the Mo microelectrode were involved in plasma ignition and collisional excitation of atoms, thus generating spectral lines with excitation energy below 7.6 eV.

### 3.3. Limits of Detection by SSETV-µCCP-OES and Comparison with Other Methods

According to Table 2, the LODs in liquid sample for SSETV-µCCP-OES method using the Maya2000 Pro microspectrometer and peak area of transient episodic signals for the most sensitive lines were as follows (ng mL^−1^): 14–As, 15–Bi, 8–Sb, 13–Se, 30–Te, 0.7–Hg, 5–Pb, 3–Sn. The corresponding limits in solid were as follows (mg kg^−1^): 0.35–As, 0.37–Bi, 0.20–Sb, 0.33–Se, 0.75–Te, 0.02–Hg, 0.13–Pb, and 0.08–Sn. It should be observed an enhancement of 2–20 folds of LODs by using the Maya2000 Pro microspectrometer compared to the QE65 Pro used in this study and previously [63,64,65,66]. The very good detection limits achieved for these elements, even avoiding chemical vapor generation, were due to the higher sensitivity of the Maya2000 Pro microspectrometer (Table 1) but also to the high stability of plasma discharge. Thus, the percentage relative standard deviation of background from the first 10 episodes (1 s integration time) was in the range 0.4–1.3%. The determination coefficients of the calibration curves up to 5 µg mL^−1^ element and measurements at the most sensitive lines were better than 0.9991. The best LODs were achieved for Hg, Pb, and Sn, which were contaminants of concern for environment. The explanation is the low excitation energy corresponding to the resonance or non-resonance atomic lines (4.89 eV for Hg 253.652 nm, 5.71 eV for Pb 261.418 nm and Pb 280.199 nm, 4.38 eV for Pb 368.346 nm, 4.87 eV and 5.97 eV for Sn 326.233 nm and Sn 207.307 nm). The LODs estimated using the maximum height of the transient peak (Table 3) were poorer than those obtained for the peak area. Getting the maximum height of the transient emission signal was simpler, but the measurement repeatability was not as good. This happened because the maximum signal height depended much more on the temporal vaporization of the sample and affected the parameters of the calibration curve (R^2^, s_y/x_). Another cause was the low vaporization temperature and thus slow sample vaporization, which resulted in the broadening of the transient signal of elements. For several spectral lines, the calibration curves for measurements with the QE65 Pro microspectrometer had a very low slope and a poor linearity, because of the low signals, and they were omitted.

Compared to other microplasma sources and microsample introduction by ETV used in OES analysis, several remarks can be made. The absolute LOD of 50 pg Pb at the spectral line 261.417 nm in the SSETV-µCCP-OES method was better than that obtained in an Ar–H_2_ microplasma of lower power (3 W instead of 15 W) and lower sample volume (3 µL instead of 10 µL) [59]. The LODs for Hg, Pb, and As in the SSETV-µCCP-OES method were poorer than those obtained by ETV-DBD-OES (0.4; 8.95 and 11.65 ng mL^−1^), because the dielectric barrier discharge was operated at higher power (37 W) and higher Ar flow (300 mL min^−1^) [62]. However, our studies have resulted in expanding the applicability of microplasmas to the determination of at least Sb, Se, Te and Sn without hydride generation. Compared to ETV-AFS, our SSETV-µCCP-OES method provided better LODs (ng mL^−1^) for Se (30/13), Sn (20/3), similar for As (10/14), Pb (3/5), and poorer for Bi (2/15) and Te (3/30) [49]. Against the classical ICP OES with pneumatic nebulization, our LODs were better for Hg, Pb, Sb, and Sn, and poorer for As [66]. The detectability of As, Sb, Se, and Te in our method was poorer than in SSETV-ICP-MS in coal analysis [50]. Furthermore, the LODs of As and Se were poorer than in GFAAS [40], while the LOD of As was poorer than in HR-CS-GFAAS with direct solid sampling of soil [43]. Compared to CVG-HR-CS-GFAAS with in situ hydride trapping, LODs for As, Bi, and Sb were poorer, while that for Hg was quite similar [20,42]. In this comparative evaluation of the detection limits, the benefits of the miniature SSETV-µCCP-OES instrumentation were associated to low cost, and the low power/low Ar consumption of plasma microtorch should not be ignored. These features make the difference from the classical ICP OES and ICP-MS laboratory instruments highly sensitive; however, they are fairly expensive in themselves and through the high consumption of energy and gases. On the other hand, compared to GFAAS with line sources/continuous Xe lamp and direct solid/liquid sampling and derivatization, the SSETV-µCCP-OES method has the advantage of simultaneous quantification avoiding derivatization to chemical vapor. This substantially simplifies the method without significantly worsening the LODs.

### 3.4. Accuracy of the SSETV-µCCP-OES Method and Non-Spectral Interferences

Data for the analysis of CRMs of soil and water sediment (Table 4 and Table 5) by the SSETV-µCCP-OES method showed recovery of analytes in the range of 92–108% for measurements using the standard addition and 86–116% for external calibration. In both cases, the confidence interval for the pooled recovery for each analyte encompassed 100%, so the differences between the two approaches were considered random. In the case of elements not certified in CRMs (Te) or for which a single CRM was available (Bi), the accuracy was checked by analyzing fortified samples spiked up to 2 µg mL^−1^ element. Recoveries achieved using external calibration were in the range 92–110%. Individual recoveries of added concentration were 102 ± 10% Teand 101 ± 3% Bi. These results demonstrated that the vaporization of the microsample at 1300 °C, rigorously adjusted, led to the separation of As, Bi, Sb, Se, Te, Hg, Pb, and Sn from the sample matrix, thus avoiding the non-spectral interference (Supplementary Materials Tables S1 and S2). Under these circumstances, the external calibration was used for quantification instead of the tedious, time-consuming standard addition method.

### 3.5. Analysis of Cave and River Sediment Samples by SSETV-µCCP-OES Method

The data in Table 6 show a repeatability of 1.2–9.9% (*n* = 5) for the measurements of As, Bi, Sb, Se, Te, Hg, Pb, and Sn in river and cave sediment by external calibration. The concentrations decreased in the following order: Pb > Bi > Te > Sb ≈ Se ≈ Sn >> Hg. Some samples exhibited extreme concentrations, i.e., about 588 mg kg^−1^ Pb and 130 mg kg^−1^ Bi. In the river sediment collected near a former chlor-alkali plant, Hg concentration was by one order of magnitude higher than in the other samples (15–31 mg kg^−1^). Bismuth was also found in higher concentrations in these samples (94–136 mg kg^−1^), while Pb was below the limit of detection (0.13 mg kg^−1^).

## 4. Materials and Methods

### 4.1. Instrumentation

**Figure 6:**
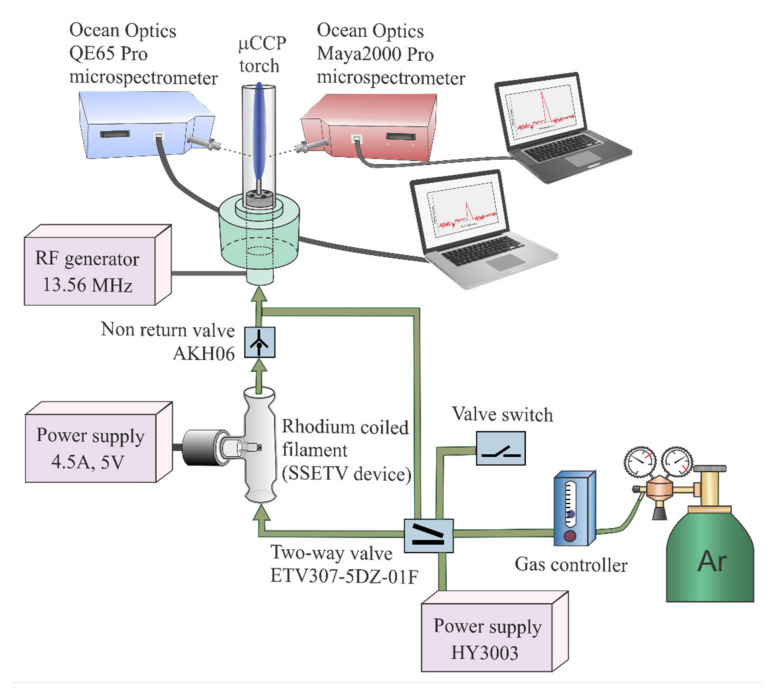
The fully miniaturized SSETV-µCCP-OES analytical system.

The prototype experimental set-up (Figure 6) for SSETV-µCCP-OES used in this study was previously described [63,64,65,66,67] and patented [69]. The analytical system is fully miniaturized and its constructive features and operation conditions are given in Table 7. The difference from previous studies was that two microspectrometers, Maya2000 Pro and QE65 Pro (Dunedin, FL, USA), were used simultaneously in plasma emission measurements.

The operating sequence of the SSETV-µCCP-OES set-up was as follows. The Rh filament was extracted from the vaporization chamber by moving back the plunger. A volume of 10 μL sample was deposited on the filament with a Hamilton syringe and heated for 180 s in air at 80 °C. The temperature was controlled by adjusting the voltage and current passing through the filament based on a previously established relationship between electrical resistance and filament temperature. The IR Optris 3 ML Optris GmbH thermometer (Berlin, Germany) was used for the measurement of temperature in this step. During sample drying, the Ar flow was directed into the plasma microtorch, bypassing the vaporization chamber via the two-way valve. In the next step, the dried sample was vaporized, and 3D spectral episodes (emission intensity–wavelength–time) were recorded in the High-Speed Acquisition mode of the Spectrasuite software. For this, the Rh filament was reinserted in the vaporization chamber and heated for 10 s at 1300 °C. The temperature was controlled based on the electrical resistance–temperature relationship applicable in the range 800–1700 °C. Measurements were performed with the IR Optris 1 MH-CF3 thermometer, Optris GmbH (Berlin, Germany). In this stage, the Ar flow was redirected through the vaporization chamber. As the vaporization of the microsample occurred, the vapor was transported into the plasma by the Ar flow, and recording of the 3D episode spectra was started. The Play Back option of the spectrometer software facilitated the visualization of each episode and finding in which the maximum signal occurred. The signal emission was assessed in two ways, namely, peak area obtained by summation of the net episodic signals that resulted after background correction using the two-point approach [65,66] and maximum height of the transient emission. The Rh filament was cleaned by heating to 1600 °C for 10 s and observation for emission in the Play Back mode.

### 4.2. Reagents and Solutions

The following reagents purchased from Merck (Darmstadt, Germany) were used: nitric acid 65% (m/m) ultrapure, HCl 35% (m/m) ultrapure, H_2_O_2_ 30% (m/m) pro-analysis, KBr suprapure, and KBrO_3_ pro-analysis. Single element standard solutions of 1000 µg mL^−1^ were used for the preparation of standards for external calibration over the range 0–5 µg mL^−1^ As, Bi, Sb, Se, Te, Sn, and Pb, and 0–1 µg mL^−1^ Hg (*n* = 10). In the standard addition approach, aliquots of up to 0.9 mL solution of CRM or the test sample were spiked at three levels to provide up to 2 µg mL^−1^ As, Bi, Sb, Se, Te, Pb, and Sn, and up to 0.5 µg mL^−1^ Hg to a final dilution of 1 mL. A multielement solution containing 3 µg mL^−1^ As, Bi, Se, Te, and Sn, 1 µg mL^−1^ Pb and Sb, and 0.2 µg mL^−1^ Hg was used for the optimization of the operating conditions of the SSETV-µCCP-OES analytical system and identifying the emission lines of elements in the spectrum. Blank solution of aqua regia was analyzed for testing the purity of chemical reagents used for the preparation of samples and calibration standards. All glassware and digestion vessels were daily cleaned by filling with a solution prepared by dissolving 1.5 g KBr and 1.08 g KBrO_3_ in 100 mL concentrated HCl and further diluted 1:10. Ultrapure water (18 MΩ cm) prepared in the laboratory with the Milli-Q system (Bedford, USA) was used throughout the study.

### 4.3. Certified Reference Materials and Test Samples

The following certified reference materials (CRMs) were analyzed to check the accuracy of the SSETV-µCCP-OES method: BCR-280 R Lake sediment, ERM-CC580 Estuarine Sediment, ERM-CC141 Loam soil, BCR-142 R Light sandy soil, BCR-287 A Thermally refined lead from the Institute for Reference Materials and Measurements–IRMM (Geel, Belgium), NC SDC 78301, LGC 6141 Soil Contaminated with Clinker Ash (Department of Trade and Industry, Teddington Middlesex, UK), AP-Metranal 32 Light sandy soil, elevated analyte levels, AP-Metranal 34 Loam metals from Analytika Spol (Vysocany, Czech Republic), CRM025–050 Metals in soil (Resource Technology Corporation, Laramie, WY, USA), CRM048–50 G Sandy soil, and SQC-001-30 G Loamy clay (Sigma Aldrich RTC, Laramie, WY, USA).

The applicability of the proposed method was verified by analyzing 14 test samples, namely, 11 sediments collected from several caves in Romania: Lesu (Bihor), Movile (Constanta), Muierilor (Women’s) (Gorj), Topolnita (Mehedinti), and 3 river sediments collected from the Aries River, in the vicinity of a former chemical plant (Turda).

The analyte concentrations were determined by both external calibration and standard addition from 5 parallel measurements.

### 4.4. Sites Description and Sampling

A brief description of the caves and river where the sediment samples were collected is presented in the Supplementary Materials. The location of the sites on the map of Romania is presented in Figure S2.

### 4.5. Sample Preparation and Analytical Procedure

Approximately 200 g test samples were dried at 100 ± 5 °C in an oven for up to 4 h, minced, and sieved to <100 µm. The resulted powder was kept in brown glass containers until preparation for analysis. Amounts of 0.5–1 g CRMs of soil, water sediment, and refined Pb, and sediment test samples were mineralized in 12 mL aqua regia using the microwave digester Berghof MW3 S+ (Berghof, Germany) following an earlier protocol used by Frentiu et al. [63,64,65,66,67]. The digest was diluted to 25 mL with ultrapure water and filtered (0.45 µm pore size). The filtrate was analyzed for the determination of elements using both external calibration and standard addition. The procedure in the standard addition method is presented in the section *Reagents and solutions*.

## 5. Conclusions

It has been demonstrated that a fully miniaturized set-up consisting of a low-power and low-Ar consumption microtorch interfaced with microspectrometers is suitable for the simultaneous determination of As, Bi, Sb, Se, Te, Hg, Pb, and Sn in liquid microsamples with complex matrices. The analytical performances of the new method were investigated using the SSETV-µCCP-OES prototype system developed in our laboratory. The direct liquid microsampling was performed by the controlled heating of the Rh filament and allowed the removal of the non-spectral interferences arising from the mineral matrix of the environmental samples. Under these circumstances, the analytes could be accurately determined using external calibration, instead of the tedious standard addition. Although no chemical derivatization was used, the proposed method offered detection limits of ng mL^−1^ or less than 1 mg kg^−1^, which extended the applicability of miniaturized plasma instrumentation. The emission spectrum of the target analytes was simple and contained resonance and non-resonance lines with excitation energies in the range 4–7.6 eV. Therefore, the interfacing of low-power plasma with low-resolution microspectrometers (0.35 nm FWHM) was opportune. At the same time, the method was found to be versatile enough, since it allowed choosing several analytical lines for an element, even in the range 180–210 nm. Overall, the strengths of the cost-effective method are related to simplicity, high-throughput for simultaneous analysis, lack of non-spectral interference, fewer operational parameters to optimize by avoiding the derivatization step, and easy interfacing of miniaturized components without loss of sensitivity. These relevant features make the method attractive to the general interest. Although primarily intended for our own miniaturized instrumentation, it could be implemented on any instrument with microplasma source or even classical ICP OES, if a miniaturized electrothermal vaporization device is available. The optimization of the operating conditions of other experimental set-ups should be taken into consideration to achieve the revealed advantages in this study.

## Figures and Tables

**Table 1:** Characteristics of the lines identified in the emission spectrum recorded with Maya2000 Pro and QE65 Pro spectrometers.

Element/Line Type	λ (nm)	Excitation Energy (eV)	Relative Intensity (%)	
			Maya2000 Pro	QE65 Pro ^1^
As(I) R	189.042	6.56	38	-
	193.759	6.40	32	4
	197.262	6.28	25	5
	199.113	7.54	13	4
	200.334	7.54	34	3
	228.812	6.77	100	4
Bi(I)	196.005	6.33	9	3
	206.163	6.13	37	1
	223.060	5.56	100	3
	227.656	5.44	12	2
	289.796	5.69	59	3
	293.830	6.13	43	2
	299.333	5.56	21	2
	302.462	6.01	47	2
	303.487	-	19	3
Sb(I) R	206.833	5.99	46	3
	217.581	5.70	80	2
	231.147	5.36	66	2
	252.852	6.12	100	3
	326.751	5.83	-	1
	338.315	5.70	-	1
Se(I)	196.090	6.32	100	3
	203.985	6.32	34	3
	207.479	5.97	10	9
Te(I)	182.215	6.81	8	-
	185.201	7.28	5	-
	199.484	6.80	6	4
	238.579	5.78	100	2
Hg(I) R	184.950	6.70	10	-
	253.652	4.89	100	5
Pb(I) R	217.000	5.71	33	3
	261.418	5.71	100	3
	280.199	5.71	78	3
	363.957	4.38	-	1
	368.346	4.38	-	1
Sn(I)	207.307	5.97	100	4
	326.233	4.87	-	8

^1^ Relative intensity compared to that observed with Maya2000 Pro.

**Table 2:** Parameters of the calibration curves, LODs in liquid and solid samples by the SSETV-µCCP-OES method using Maya2000 Pro and QE65 Pro microspectrometers for peak area measurement of the transient signals.

Element	λ (nm)	Maya2000 Pro					QE65 Pro				
		Slope (mL µg^−1^)	R^2^	s_y/x_ (a.u)	LOD in Liquid (ng mL^−1^)	LOD in Solid ^1^ (mg kg^−1^)	Slope (mL µg^−1^)	R^2^	s_y/x_ (a.u)	LOD in Liquid (ng mL^−1^)	LOD in Solid ^1^ (mg kg^−1^)
As	189.042	21398	0.9999	100	14	0.35	-	-	-	-	-
	193.759	18020	0.9995	150	25	0.63	649	0.9994	8	40 ^2^	1.00 ^2^
	197.262	12824	0.9992	130	30	0.75	641	0.9993	10	49	1.22
	200.334	18188	0.9996	120	20	0.50	543	0.9991	10	60	1.50
Bi	223.060	60608	0.9998	300	15	0.37	1560	0.9987	50	100	2.50
	293.830	35759	0.9998	300	25	0.63	-	-	-	-	-
	302.462	28486	0.9997	310	33	0.82	-	-	-	-	-
Sb	206.833	103961	0.9983	620	18	0.45	1789	0.9980	30	50	1.25
	217.581	116758	0.9987	683	17	0.43	2816	0.9992	38	40 ^2^	1.00 ^2^
	252.852	145462	0.9994	385	8	0.20	4623	0.9993	40	30	0.75
Se	196.026	22186	0.9999	100	13	0.33	1024	0.9991	52	152	3.75
	203.985	6878	0.9991	100	44	1.10	-	-	-	-	-
Te	182.215	4567	0.9997	385	140	3.50	-	-	-	-	-
	238.579	79690	0.9998	833	30	0.75	1660	0.9970	277	501	12.52
Hg	253.652	981663	0.9999	230	0.7	0.02	34235	0.9996	171	15 ^2^	0.37 ^2^
Pb	261.417	170830	0.9998	289	5	0.13	4506	0.9994	45	30	0.75
	280.199	132913	0.9998	270	6	0.15	3418	0.9994	30	30	0.75
	217.000	57162	0.9992	520	27	0.67	1607	0.9991	54	101	2.53
	368.346	-	-	-	-	-	2431	0.9997	16	20 ^2^	0.50 ^2^
Sn	207.307	8116	0.9992	8	3	0.08	300	0.9990	1	10	0.25
	326.233	-	-	-	-	-	2727	0.9999	4	4.4 ^2^	0.11 ^2^

^1^ Limits of detection in solid calculated for 1.0000 g digested sample made up to 25 mL. ^2^ Limits of detection provided in reference [66].

**Table 3:** Parameters of the calibration curves and LOD in liquid and solid samples by the SSETV-µCCP-OES method using Maya2000 Pro and QE65 Pro microspectrometers for peak height measurement of transient signals.

Element	λ (nm)	Maya2000 Pro					QE65 Pro				
		Slope (mL µg^−1^)	R^2^	s_y/x_ (a.u)	LOD in Liquid (ng mL^−1^)	LOD in Solid ^1^ (mg kg^−1^)	Slope (mL µg^−1^)	R^2^	s_y/x_ (a.u)	LOD in Liquid (ng mL^−1^)	LOD in Solid ^1^ (mg kg^−1^)
As	189.042	682	0.9993	16	70	1.75	-	-	-	-	-
	193.759	589	0.9990	18	90	2.25	-	-	-	-	-
	197.626	500	0.9984	16	96	2.40	-	-	-	-	-
	200.334	668	0.9991	18	80	2.00	-	-	-	-	-
Bi	223.060	7032	0.9994	70	30	0.75	153	0.9989	6	124	3.10
	293.830	2807	0.9993	47	50	1.25	-	-	-	-	-
	302.462	1005	0.9993	25	75	1.88	-	-	-	-	-
Sb	206.833	3320	0.9979	34	31	0.78	-	-	-	-	-
	217.581	3638	0.9982	35	29	0.73	-	-	-	-	-
	252.852	4322	0.9984	25	17	0.43	149	0.9954	10	200	5.00
Se	196.026	1970	0.9991	46	70	1.75	-	-	-	-	-
	203.985	1069	0.9981	36	100	2.50	-	-	-	-	-
Te	182.215	299	0.9869	33	330	8.25	-	-	-	-	-
	238.579	3689	0.9972	69	56	1.40	100	0.9888	15	450	11.25
Hg	253.652	58955	0.9987	197	10	0.25	2165	0.9905	50	70	1.80
Pb	217.000	6701	0.9952	114	51	1.28	137	0.9907	10	220	5.50
	261.417	21508	0.9957	143	20	0.50	502	0.9913	14	80	2.00
	280.199	17586	0.9961	170	29	0.73	384	0.9944	12	90	2.25
	368.346	-	-	-	-	-	260	0.9975	14	160	4.00
Sn	207.307	450	0.9985	6	40	1.00	12	0.9932	1	250	6.25
	326.233	-	-	-	-	-	323	0.9947	15	140	3.50

^1^ Limits of detection in solid calculated for 1.0000 g digested sample made up to 25 mL.

**Table 4:** Results for the analysis of soil and water sediment CRMs by the SSETV-µCCP-OES method using Maya2000 Pro microspectrometer for As, Hg, Pb, and Sb using standard addition and external calibration.

CRM	Certified Value ± U (mg kg^−1^) ^1^				Found Value ± CI (mg kg^−1^) ^2^ Standard Addition				Found Value ± CI (mg kg^−1^) ^2^ External Calibration			
	As	Hg	Pb	Sb	As	Hg	Pb	Sb	As	Hg	Pb	Sb
ERM–CC141	7.5 ± 1.4	0.08 ± 0.008	-	-	8.0 ± 1.5	0.08 ± 0.009	-	-	7.9 ± 1.5	0.07 ± 0.005	-	-
CRM04850 G	123 ± 3.4	28 ± 1.13	86.9 ± 2.42	139 ± 13.9	122 ± 4.3	28 ± 1.60	86.9 ± 2.45	142 ± 14.1	124 ± 4.3	29 ± 1.62	84.9 ± 2.5	138.5 ± 14.0
LGC6141	13.2 ± 3.5	-	75.8 ± 16	-	12.1 ± 1.1		73.9 ± 2.30	-	13.8 ± 0.8	-	75.8 ± 3.65	-
Metranal–32	26.1 ± 1.1	0.120	35.5 ± 0.9	-	25.6 ± 1.1	0.13 ± 0.005	36.2 ± 2.5	-	25.7 ± 1.4	0.12 ± 0.01	33.4 ± 2.4	-
Metranal–34	42.4 ± 2.2	0.21	83.1 ± 2.3	-	43.0 ± 2.4	0.20 ± 0.012	82.6 ± 3.5	-	44.5 ± 2.3	0.22 ± 0.02	82.8 ± 2.7	-
BCR–142 R	-	-	25.7 ± 1.6	-	-	-	25.8 ± 2.16	-	-	-	25.8 ± 1.7	-
BCR–287 A	-	-	-	0.04 ± 0.015	-	-	-	<0.20	-	-	-	<0.20
NC SDC 78301	56 ± 10	0.22 ± 0.04	79 ± 12	-	55 ± 10	0.21 ± 0.05	79 ± 13	-	55 ± 12	0.19 ± 0.04	82 ± 13	-
ERM–CC580	-	132 ± 3	-	-	-	128 ± 8	-	-	-	125 ± 4	-	-
CRM025	339 ± 20	99.8 ± 18	1447 ± 88	<3.2	341 ± 20	104 ± 19	1366 ± 93	3.1 ± 0.2	357 ± 21	94.9 ± 18	1370 ± 92	3.5 ± 0.4
BCR–280 R	33.4 ± 2.9	1.46 ± 0.2	-	-	35.0 ± 3.7	1.45 ± 0.3	-	-	29.6 ± 3.1	1.26 ± 0.2	-	-
RTCSQC001	43.1 ± 0.7	2.86 ± 0.1	144 ± 2	42.0 ± 4.1	45.2 ± 2.0	3.02 ± 0.2	134 ± 5	43.1 ± 4.7	44.2 ± 0.9	2.99 ± 0.1	139 ± 3	48.9 ± 4.2
Rec. range (%)					92–107	95–108	93–102	97–103	89–105	86–105	94–104	100–116
Pooled rec. (%)					101 ± 10	101 ± 13	98 ± 8	101 ± 9	101 ± 10	96 ± 12	98 ± 8	108 ± 10

^1^ U is the expanded uncertainty for 95% confidence level (k = 2). ^2^ CI is the confidence interval for 95% confidence level (*n* = 5).

**Table 5:** Results for the analysis of soil and water sediment CRMs by SSETV-µCCP-OES method using Maya2000 Pro microspectrometer for Bi, Se, Te, and Sn and standard addition and external calibration.

CRM	Certified Value ± U (mg kg^−1^) ^1^				Found Value ± CI (mg kg^−1^) ^2^ Standard Addition				Found Value ± CI (mg kg^−1^) ^2^ External Calibration			
	Bi	Se	Te	Sn	Bi	Se	Te	Sn	Bi	Se	Te	Sn
CRM048	-	178 ± 5.68	-	93.5 ± 3.26	-	176 ± 5.91	-	96.6 ± 3.26	-	176 ± 6.10	-	95.4 ± 6.07
BCR 142 R	-	-	-	-	-	-	-	-	-		-	-
BCR 287	67.3 ± 1.1	-	-	-	67.3 ± 4.6	-	-	-	68.0 ± 5.3		-	-
NC SDC 78301	-	0.39 ± 0.1	-	-	-	0.35 ± 0.1	-	-	-	0.40 ± 0.1	-	-
CRM025	-	518 ± 31	-	-	-	536 ± 38	-	-	-	550 ± 32	-	-
SQC001	-	154 ± 3	-	215 ± 8	-	156 ± 7	-	212 ± 12	-	148 ± 7	-	203 ± 9
Rec. range (%)					94–107	90–103	-	99–103	98–104	96–106	-	94–102
Pooled rec. (%)					100 ± 7	98 ± 15	-	101 ± 5	101 ± 3	101 ± 13	-	98 ± 3

^1^ U is the expanded uncertainty for 95% confidence level (k = 2). ^2^ CI is the confidence interval for 95% confidence level (*n* = 5).

**Table 6:** Results for As, Bi, Sb, Se, Te, Hg, Pb, and Sn in test samples of cave and river sediment by the SSETV-µCCP-OES method using Maya2000 Pro microspectrometer, and external calibration.

Sample Origin	Sample Code	Concentration ± CI (mg kg^−1^) ^1^							
		As	Bi	Sb	Se	Te	Hg	Pb	Sn
Lesu Cave	1	<0.35	20.86 ± 0.53	7.02 ± 0.28	<0.33	8.38 ± 0.18	0.23 ± 0.01	588.19 ± 21.52	6.77 ± 0.33
	2	1.52 ± 0.05	36.90 ± 2.30	1.20 ± 0.06	<0.33	5.37 ± 0.13	0.27 ± 0.01	10.29 ± 0.44	9.63 ± 0.19
Movile Cave	3	<0.35	107.34 ± 2.65	6.53 ± 0.26	6.07 ± 0.27	8.49 ± 0.15	0.17 ± 0.01	16.13 ± 0.41	5.94 ± 0.30
Muierilor Cave	4	1.72 ± 0.04	57.79 ± 1.91	2.61 ± 0.04	3.39 ± 0.13	5.93 ± 0.30	1.49 ± 0.04	83.25 ± 4.26	4.83 ± 0.17
	5	2.20 ± 0.06	130.87 ± 6.85	37.19 ± 3.06	11.91 ± 0.74	11.54 ± 0.59	0.11 ± 0.01	76.87 ± 3.60	3.97 ± 0.09
	6	1.88 ± 0.15	54.78 ± 3.97	16.95 ± 0.19	1.11 ± 0.05	36.29 ± 2.90	0.22 ± 0.01	1.74 ± 0.06	4.67 ± 1.34
	7	6.10 ± 0.17	63.13 ± 2.55	22.40 ± 1.38	25.76 ± 0.91	9.68 ± 0.37	0.21 ± 0.02	67.83 ± 2.68	4.52 ± 0.15
	8	1.08 ± 0.03	7.66 ± 0.21	<0.20	<0.33	4.42 ± 0.16	0.28 ± 0.01	7.70 ± 0.45	3.51 ± 0.14
	9	1.54 ± 0.03	12.97 ± 0.26	3.34 ± 0.10	3.43 ± 0.14	4.94 ± 0.23	0.26 ± 0.02	10.43 ± 0.32	2.58 ± 0.12
Topolnita Cave	10	1.23 ± 0.02	45.20 ± 0.72	19.44 ± 0.75	5.24 ± 0.24	6.18 ± 0.25	0.26 ± 0.01	12.46 ± 0.59	4.62 ± 0.18
	11	0.81 ± 0.02	9.79 ± 0.26	<0.20	<0.33	2.75 ± 0.17	0.14 ± 0.01	<0.13	2.10 ± 0.11
Aries River	12	5.39 ± 0.13	129.51 ± 7.55	<0.20	0.87 ± 0.03	65.92 ± 3.07	30.70 ± 0.79	<0.13	1.64 ± 0.12
	13	5.02 ± 0.22	135.98 ± 8.72	2.38 ± 0.06	2.09 ± 0.07	<0.75	30.33 ± 1.55	<0.13	2.00 ± 0.12
	14	1.74 ± 0.08	94.19 ± 4.36	<0.20	<0.33	<0.75	15.45 ± 0.82	<0.13	0.97 ± 0.03
	RSD (%) ^2^	1.2–9.3	1.6–8.3	1.3–9.4	1.3–7.1	2.0–9.1	2.8–9.9	2.6–6.4	1.3–8.1

^1^ CI is the confidence interval for 95% confidence level (*n* = 5). ^2^ RSD is % relative standard deviation (*n* = 5 parallel measurements).

**Table 7:** Constructive characteristics and operation conditions of the SSETV-µCCP-OES analytical system equipped with two low-resolution microspectrometers.

Component	Description
Plasma microtorch	Capacitively coupled; Mo tip microelectrode, 1.25 mm diameter (Goodfellow Cambridge, UK) mounted in a quartz tube (5 mm i.d, 25 mm length, 160 nm cut-off, H. Baumbach & Co. Ltd., Ipswich, UK); intake of Ar (100–200 mL min^−1^) transporting the vaporized microsample through four holes with a diameter of 750 µm drilled in the PTFE support around the microelectrode [63,69], (Babes-Bolyai University Cluj-Napoca, Romania)
Plasma power supply	Miniaturized r.f. generator (15 × 17 × 24 cm^3^), 13.56 MHz, 10–30 W (Technical University, Cluj-Napoca, Romania)
Small-sized electrothermal vaporizer	Rh filament (99.9% purity, 250 µm diameter, 4 turns with 1.5 mm diameter), mounted in a T-shaped vaporization chamber of quartz, sample volume: 10 µL, drying temperature: 80 °C for 180 s, vaporization temperature: 1300 °C for 10 s (Babes-Bolyai University Cluj-Napoca, Romania), 5–10% precision in temperature control [63,66,69].
Power source for the Rh filament heating	Built in laboratory (Technical University, Cluj-Napoca, Romania), heating control of the Rh filament based on electrical resistance–temperature relationship [65,66].
Control of Ar flow	Two-way valve SMCEVT307–5 DZ-01 F-Q supplied by the DC power source HY3003, Mastech, Premier Farnel (Leed, UK) [65].
Optical detectors	Maya2000 Pro microspectrometer equipped with CCD (165–309 nm spectral range, 0.35 nm FWHM, detector chamber purged with Ar, Ocean Optics, Dunedin, USA).QE65 Pro microspectrometer equipped with CCD (190–380 nm spectral range, 0.4 nm FWHM, CCD cooled at −20 °C, Ocean Optics, Dunedin, USA).
Plasma viewing	Microspectrometer mounted on a 3D translator for targeting different heights by 100 µm increment, radial view through the collimating lens (10 mm focal length) without fiber optics [65,66].
Spectrum recording/emission signal measurement	70 episodic 3D spectra (signal–wavelength–time), 200 ms integration time per episode [67], measurement of the transient signal and peak height.

## Data Availability

The data presented in this study is available on request from the corresponding author.

## Supplementary Materials

The followings are available online, Figure S1: The 3D episodic spectra recorded in the SSETV-µCCP-OES method with the Maya2000 Pro microspectrometer in which the most intense lines of As, Bi, Sb, Se, Te, Hg, Pb, and Sn can be visualized, Figure S2: Map of the sampling site locations, Table S1: Composition of the multimineral matrix of soil and water sediment CRMs used to evaluate the accuracy of the SSETV-µCCP-OES method for the determination of As, Bi, Sb, Se, Te, Hg, Pb, and Sn, Table S2: Composition of the multimineral matrix of cave and river sediment test samples analyzed by the SSETV-µCCP-OES method for the determination of As, Bi, Sb, Se, Te, Hg, Pb, and Sn.

## Sample Availability

Not available.
