# Supplementary material for: Simultaneous Determination of As, Bi, Sb, Se, Te, Hg, Pb and Sn by Small-Sized Electrothermal Vaporization Capacitively Coupled Plasma Microtorch Optical Emission Spectrometry Using Direct Liquid Microsampling
Source: Molecules. 2021 Apr 30;26(9):2642. doi: 10.3390/molecules26092642 (PMC8124486; doi:10.3390/molecules26092642)
Supplement: Supplementary file 1 [file molecules-26-02642-s001.zip › molecules-1186897-supplementary.pdf]

Supplementary Material

Simultaneous Determination of As, Bi, Sb, Se, Te, Hg, Pb and Sn by Small-sized Electrothermal Vaporization Capacitively Coupled Plasma Microtorch Optical Emission Spectrometry Using Direct Liquid Microsampling

Simion Bogdan Angyus [1,2,3], Erika Levei [3], Dorin Petreus [4], Radu Etz [4], Eniko Covaci [1,2], Oana Teodora Moldovan [5,6], Michaela Ponta [1,2], Eugen Darvasi [1,2] and Tiberiu Frentiu [1,2,*]

[1] Babes-Bolyai University, Faculty of Chemistry and Chemical Engineering, Arany Janos 11, 400028 Cluj-Napoca, Romania; bogdan.angyus@gmail.com (S.B.A.); eniko.covaci@ubbcluj.ro (E.C.); michaela.ponta@ubbcluj.ro (M.P.); darvasi.jeno@gmail.com (E.D.)

[2] Babes-Bolyai University, Research Center for Advanced Chemical Analysis, Instrumentation and Chemometrics–Analytica, Arany Janos 11, 400028 Cluj-Napoca, Romania

[3] National Institute for Research and Development of Optoelectronics INOE 2000 INCD Bucharest, Research Institute for Analytical Instrumentation, Donath 67, 400293 Cluj-Napoca, Romania; erika.levei@icia.ro (E.L.)

[4] Technical University of Cluj-Napoca, Faculty of Electronics, Telecommunications and Information Technology, George Baritiu 26–28, 40002 Cluj-Napoca, Romania; dorin.petreus@ael.utcluj.ro (D.P.); radu.etz@ael.utcluj.ro (R.E.)

[5] Emil Racovita Institute of Speleology, Department Cluj-Napoca, Clinicilor 5, 4000006 Cluj-Napoca, Romania, oanamol35@gmail.com (O.T.M.)

[6] Romanian Institute of Science and technology, Saturn 24-26, 400504, Cluj-Napoca, Romania

[*] Correspondence: tiberiu.frentiu@ubbcluj.ro (T.F.); Tel.: +40-264-493833

This online resource contains the following data:

1. The 3D episode spectra recorded in the SSETV-μCCP-OES method (Figure S1)
2. Sites description and sampling of cave and river sediments (Figure S2)
3. The composition of the multimineral matrix in CRMs and test samples (Table S1 and Table S2)

1. The 3D episode spectra recorded in the SSETV-μCCP-OES method

Figure S1 shows the 3D episode spectra (intensity–wavelength–time) recorded in the SSETV-μCCP-OES method with the Maya2000 Pro microspectrometer under the optimal operating conditions. Spectra contain the most sensitive lines for As, Bi, Sb, Se, Te, Hg, Pb and Sn.

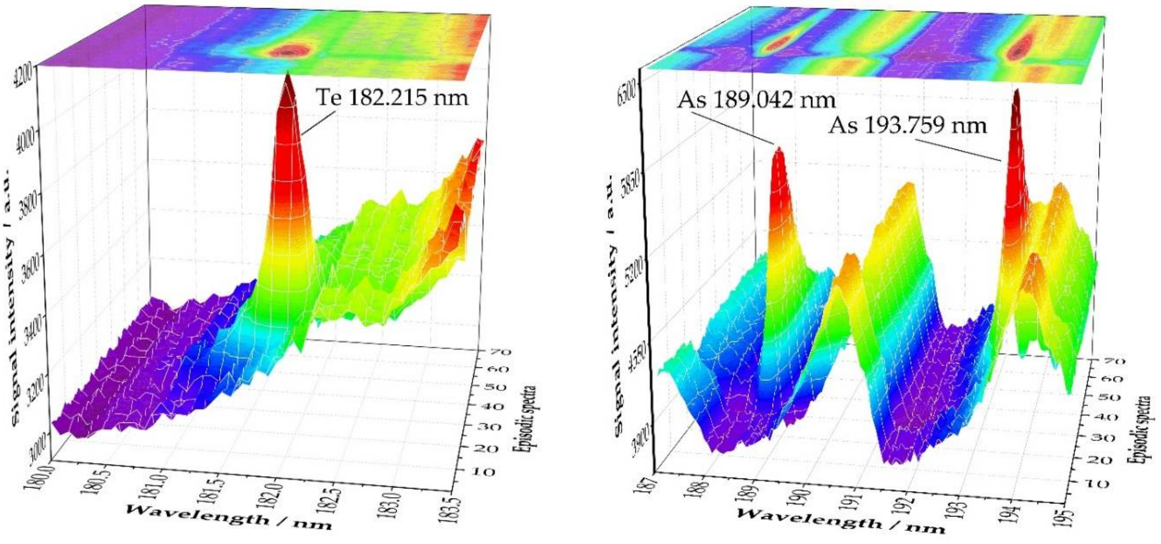

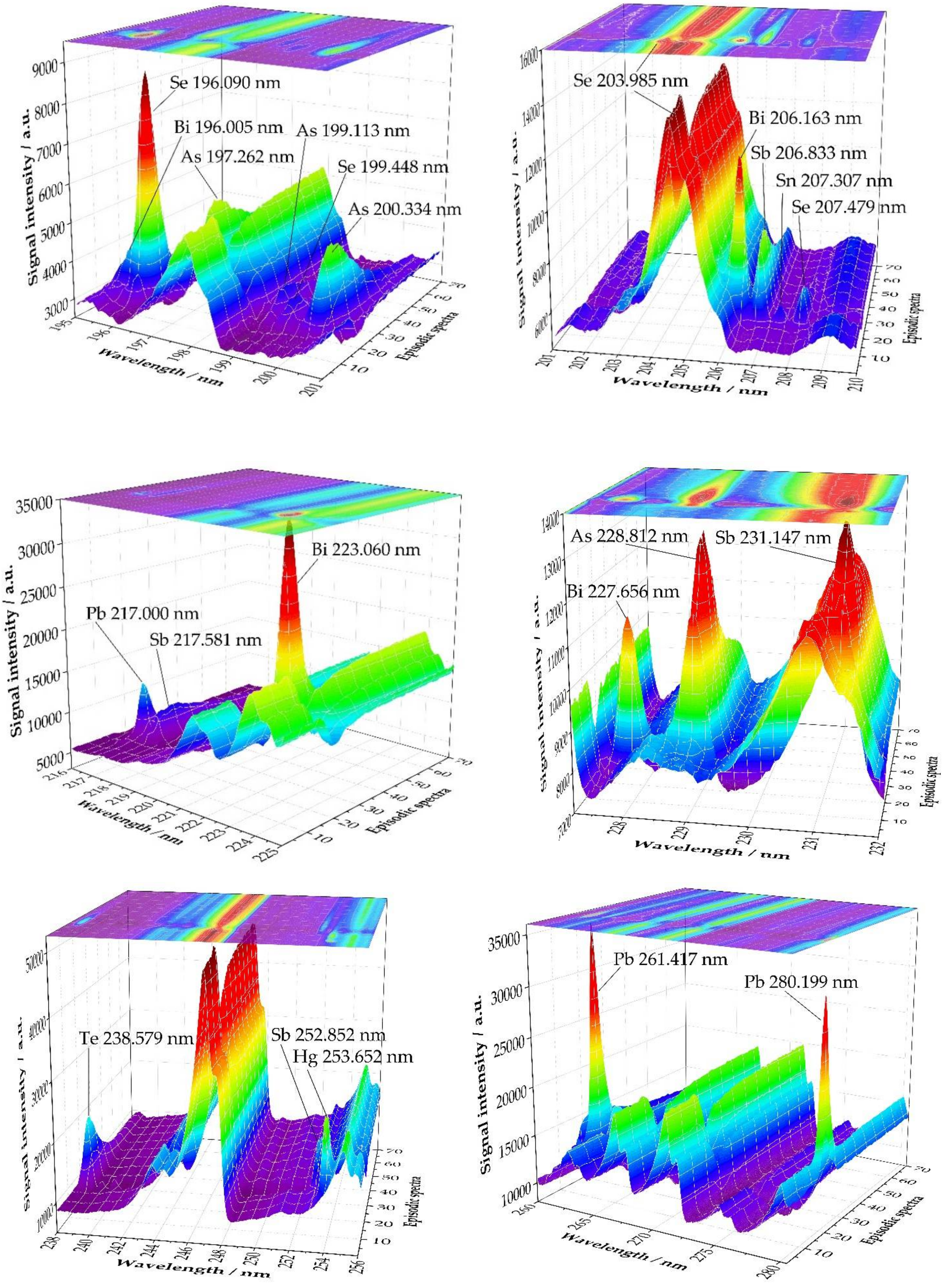

Figure S1. 3D episode emission spectra in SSETV-μCCP-OES recorded with the Maya2000 Pro microspectrometer under the optimal conditions providing the most sensitive lines of As, Bi, Sb, Se, Te, Hg, Pb and Sn.

2. Sites description and sampling

The cave floor sediment samples were collected from four caves by speleologists from the Emil Racovita Institute of Speleology, Cluj-Napoca, in 2019 and 2020. The river sediment samples were collected in 2020, from the bank of the Aries River flowing near the former Turda Chemical Plant. The sampling sites are indicated in Figure S2.

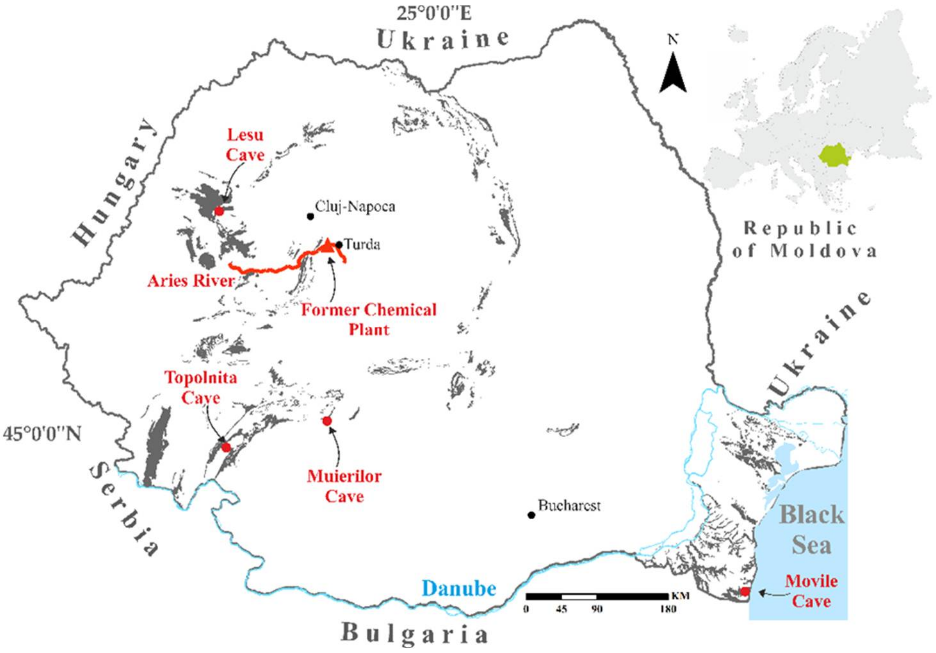

Figure S2. Map of the sampling site locations.

2.1. Lesu Cave

The cave from Lesu Valley, named here Lesu Cave, is located in Padurea Craiului Mountains (North-Western Romania) at 650 m a.s.l. and it is 800 m long. It is crossed by a stream with silty-sandy sediments, deposited along the main gallery, and few small, lateral passages. The mean annual air temperature inside the cave ranges between 8 and 10 °C. The cave was studied for microclimate, fauna and microorganisms in sediments [1–3]. Two cave sediment samples were taken from the surface of the silty/sandy sediments deposited along the stream (sample 1) and clay sediments from the bottom of a pool (sample 2).

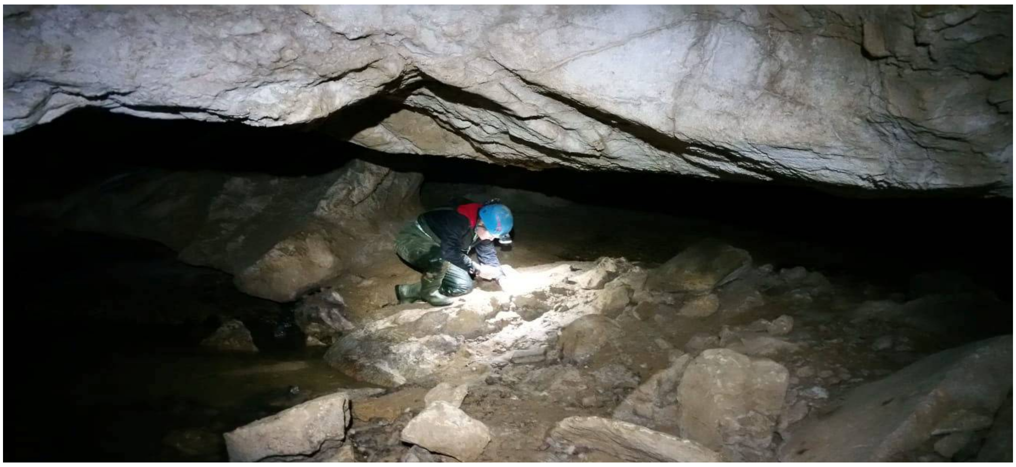

Sample collection in Lesu Cave (Copyright @ Emil Racovita Institute of Speleology, Romania).

2.2. Movile Cave

Located in Dobrogea, South-Eastern Romania, Movile Cave was discovered during geological prospection works in 1986. The artificial shaft gives access to a system of natural passages partially flooded with hydrogen sulfide-rich thermal water. Movile Cave is part of a broad underground maze of fissures and passages associated with the

sulphurous aquifer in the area [4]. The cave is developed in oolitic- and fossil-rich limestone of Sarmatian age (i.e. late Miocene, about 12.5 Ma) [5]. A sample of silty sediment (sample 3) was taken from the cave floor, near the lake with sulphurous water.

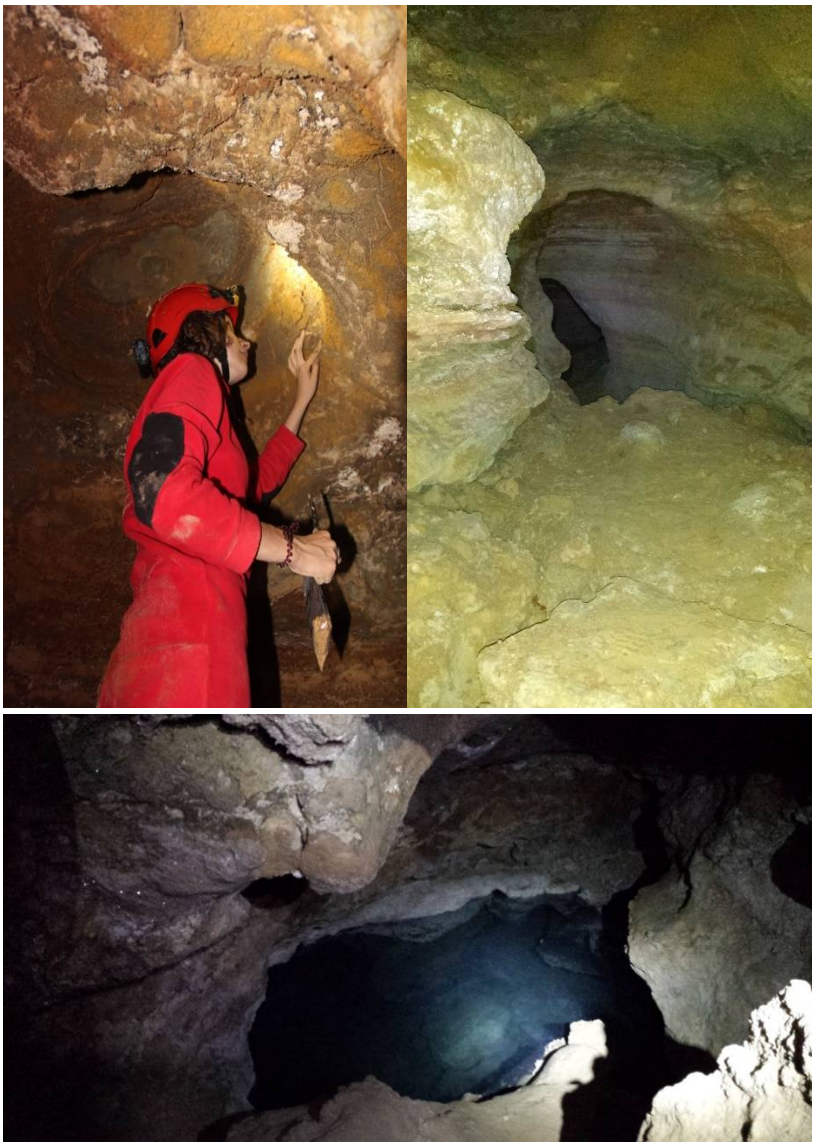

Sample collection in Movile Cave (Copyright @ Emil Racovita Institute of Speleology, Romania).

2.3. Muierilor Cave

Muierilor Cave is located in the Southern Carpathians. It is a show cave known for the discovery of early modern human remains c. 35 ka old [6,7], Paleolithic artifacts and a deposit of fossil remains of Quaternary cave mammals [8]. The cave is developed on four levels totaling more than 8 km in length. Six cave samples were taken from the cave floor and consisted of white silt covered by small limestone pebbles (sample 4), brown silt (sample 5), black clay deposit in a room with fossil guano (samples 6 and 7), and fine sediments in calcite pools (samples 8 and 9).

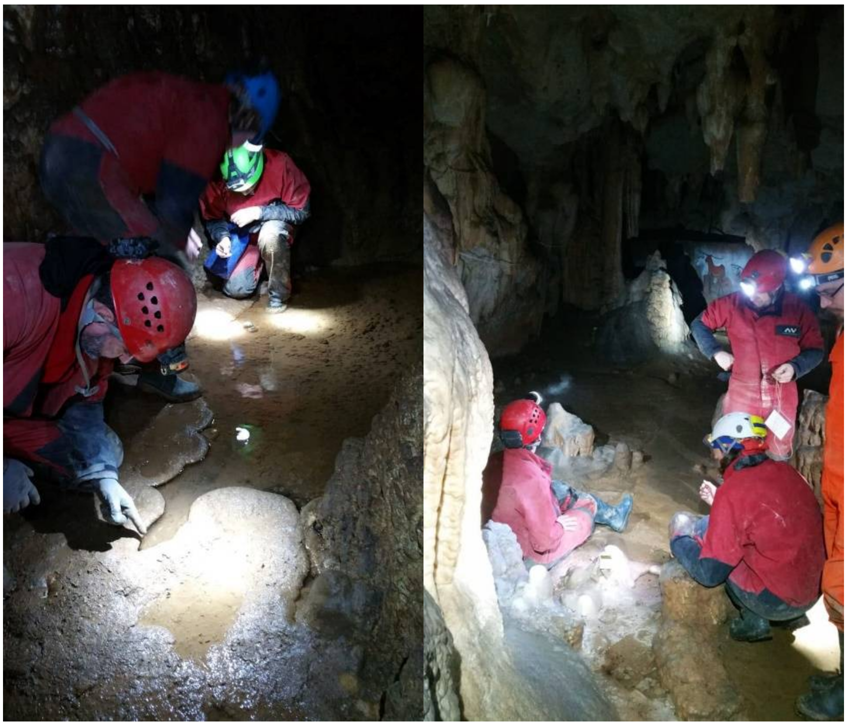

Sample collection in Muierilor Cave (Copyright @ Emil Racovita Institute of Speleology, Romania).

2.4. Topolnita Cave

Topolnita Cave belongs to a 22 km long cave system, located in Mehedinti Karstic Plateau (South-Western Romania). It is developed on multiple levels with a complex morphology, mineralogy and sedimentary deposits. The cave is known for its bat colonies and rich cave fauna [9]. Two samples of silt, deposited in a fine layer on calcite, were taken from the cave (samples 10 and 11).

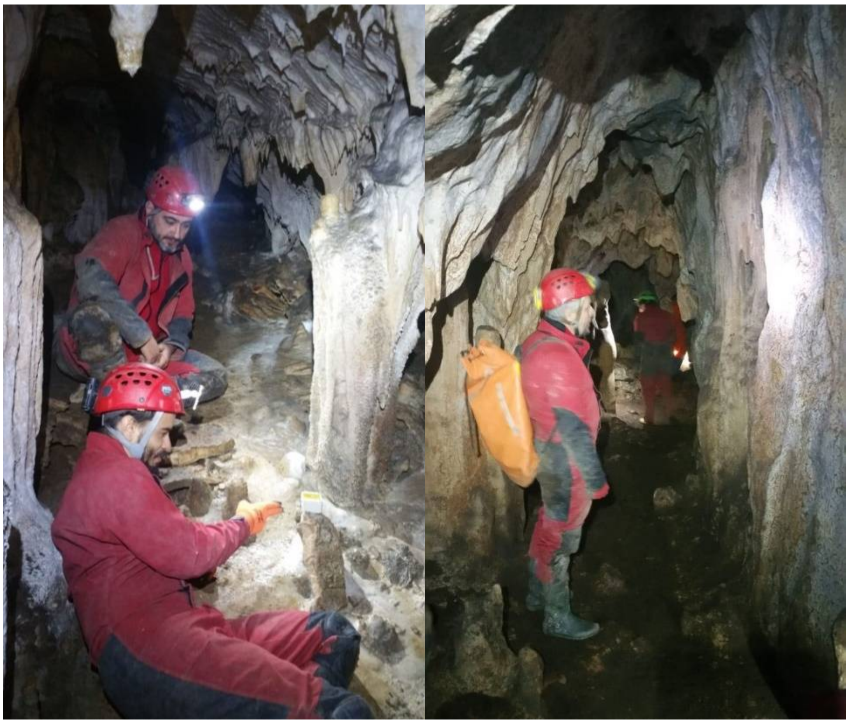

Sample collection in Topolnita Cave (Copyright @ Emil Racovita Institute of Speleology, Romania).

2.5. Turda Former Chemical Plant

Turda town is a former industrial center located in North-Western Romania (Figure S2). The former Turda Chemical Plant was the main source of income for the local economy. The plant was founded in 1911 and operated until 1998. While the chemical plant was operating, it caused soil contamination with mercury and other heavy metals, and further water pollution of the Aries River flowing nearby [10,11]. Three river sediment samples collected from the river bank, were analyzed using the SSETV-μCCP-OES analytical system (samples 12,13 and 14).

3. Composition of the multimineral matrix

The compositions of the multimineral matrix in CRMs of soil and water sediment, and test samples of cave and river sediment analyzed to evaluate the accuracy and applicability of the SSETV-μCCP-OES method for the determination of As, Bi, Sb, Se, Te, Hg, Pb and Sn are presented in Table S1 and Table S2, respectively.

Table S1. Mineral matrix composition in CRMs of soil and sediment analyzed to assess the accuracy of the SSETV-μCCP-OES method for the determination of As, Bi, Sb, Se, Te, Hg, Pb and Sn.

Sample	Concentration (μg mL^{-1})													
	**Fe**	**Ni**	**Cr**	**Co**	**Cu**	**Zn**	**Na**	**Mg**	**K**	**Ca**	**Mn**	**Ba**	**Al**	**Sr**
ERM-CC141	158.60	0.32	0.44	0.16	0.25	1.29	0.16	42.67	21.64	35.06	4.95	0.79	185.90	0.21
CRM048	66.25	0.69	1.60	1.22	1.89	4.65	36.07	40.34	44.08	38.45	6.63	1.55	99.48	1.34
LGC6141	38.02	0.42	1.15	0.94	0.53	0.65	2.32	51.02	35.83	24.64	1.24	0.44	24.70	0.14
Metranal-32	190.20	0.73	2.49	0.33	0.57	1.89	2.70	181.00	51.62	101.30	7.87	1.70	301.00	0.43
Metranal-34	220.60	0.51	0.75	0.41	3.10	3.83	1.98	86.20	48.76	138.70	10.14	3.39	416.20	1.37
BCR 142R	185.50	0.89	1.22	0.25	1.22	2.26	2.03	119.40	33.31	32.00	10.03	1.60	461.20	0.18
BCR 287	0.10	0.03	0.01	0.03	0.07	0.47	-	0.19	-	0.44	0.01	0.02	0.11	-
NCSDC78301	25.84	0.32	0.85	0.15	0.43	2.38	1.64	38.53	15.76	29.32	1.39	0.85	96.49	0.18
ERM-CC580	29.63	0.22	0.69	0.05	0.22	1.99	24.31	52.16	7.65	894.40	0.74	0.50	59.26	3.12
CRM025-050	38.58	0.09	1.51	0.05	0.10	1.48	-	14.34	5.16	85.50	0.74	7.90	37.62	1.38
BCR 280r	82.86	0.23	0.36	0.11	0.24	1.52	2.17	43.40	20.45	39.88	1.94	0.92	116.10	0.23
SQC001-30G	46.26	0.67	1.23	0.44	1.23	3.69	2.10	7.19	12.30	15.64	3.73	2.23	103.60	1.53
Minimum	0.10	0.03	0.01	0.03	0.07	0.47	0.16	0.19	5.16	0.44	0.01	0.02	0.11	0.14
Maximum	220.60	0.89	2.49	1.22	3.10	4.65	36.07	181.00	51.62	894.40	10.14	7.90	461.20	3.12
Median	56.26	0.37	1.00	0.20	0.48	1.94	2.14	43.04	21.64	36.76	2.83	1.24	101.54	0.43
Mean	90.20	0.43	1.02	0.34	0.82	2.18	7.55	56.37	26.96	119.61	4.12	1.82	158.47	0.92
St. dev.	76.7	0.3	0.7	0.4	0.9	1.3	12.3	51.1	16.6	247.2	3.7	2.1	153.4	0.9

Table S2. Mineral matrix composition in test samples of cave and river sediment used to assess the applicability of the SSETV-μCCP-OES method for the determination of As, Bi, Sb, Se, Te, Hg, Pb and Sn.

Sample	Concentration (μg mL^{-1})													
	Fe	Ni	Cr	Co	Cu	Zn	Na	Mg	K	Ca	Mn	Ba	Al	Sr
1	26.14	0.05	0.03	0.01	0.08	0.39	0.76	5.27	12.76	97.00	1.49	0.36	68.40	0.04
2	25.39	0.05	0.02	0.02	0.03	0.14	0.20	3.50	4.36	66.97	1.50	0.18	30.68	0.03
3	22.98	0.01	0.01	0.01	0.01	0.02	3.01	16.66	8.91	261.60	0.59	0.44	34.26	0.46
4	28.60	0.02	0.01	-	0.01	0.06	0.40	14.49	7.90	956.76	0.31	0.03	-	0.12
5	106.00	0.02	0.04	0.01	0.58	0.34	1.44	5.53	11.30	105.00	0.20	0.57	-	0.12
6	142.90	0.05	0.07	0.02	3.08	1.07	0.59	8.20	10.69	182.06	0.40	0.34	-	0.11
7	105.30	0.18	0.16	0.08	17.53	2.31	0.63	3.59	5.03	179.70	1.36	0.77	32.40	0.13
8	59.98	0.04	0.04	0.01	0.09	0.33	2.06	15.08	7.22	392.50	0.80	0.13	63.89	0.09
9	69.37	0.03	0.04	0.01	0.47	0.62	99.48	10.85	11.23	378.30	0.61	0.21	76.27	0.15
10	91.25	0.02	0.04	0.01	0.02	0.06	1.04	22.77	6.43	35.09	0.39	0.13	84.44	0.03
11	110.80	0.02	0.05	0.01	0.02	0.06	5.05	29.60	9.13	35.78	0.41	0.17	114.70	0.03
12	100.02	0.04	0.02	0.02	0.90	0.76	24.00	34.29	10.03	10.70	0.02	-	129.89	0.01
13	104.25	0.07	0.02	0.03	0.41	1.23	24.78	40.26	10.77	3.83	-	-	148.40	0.02
14	101.48	0.04	0.04	0.01	0.92	1.27	25.56	43.23	10.51	12.35	-	0.33	146.92	0.04
Minimum	22.98	0.01	0.01	0.01	0.01	0.02	0.20	3.50	4.36	3.83	0.02	0.03	30.68	0.01
Maximum	142.90	0.18	0.16	0.08	17.53	2.31	99.48	43.23	12.76	956.76	1.50	0.77	148.40	0.46
Median	95.64	0.04	0.04	0.01	0.25	0.37	1.75	14.79	9.58	101.00	0.50	0.27	76.27	0.07
Mean	78.18	0.05	0.04	0.02	1.73	0.62	13.50	18.09	9.02	194.12	0.67	0.31	84.57	0.10
St. Dev.	39.26	0.04	0.04	0.02	4.62	0.66	26.65	13.76	2.51	255.19	0.51	0.21	44.54	0.11

Author Contributions: Methodology, investigation, validation, S.B.A.; Funding acquisition, resources, E.L.; Software, D.P.; Software,R.E.; Formal analysis, visualization, data curation, writing—review and editing,E.C.; Funding acquisition, project administration, resources,O.T.M.; Writing—original draft,M.P.; Visualization, software,E.D.; Conceptualization, supervision, funding acquisition, writing—review and editing,T.F. All authors have read and agreed to the published version of the manuscript.

Funding: This research was funded by The Ministry of Research and Innovation, Romania, CNCS—UEFISCDI, project number PN-III-P4-ID-PCCF-2016-0016 (DARKFOOD) and project number 33PFE/2018, within PNCDI III. The APC was funded by The Ministry of Research and Innovation, Romania, CNCS—UEFISCDI, project number PN-III-P4-ID-PCCF-2016-0016

Acknowledgments: The authors would like to thank Dr. Ionuț Cornel Mirea for his help with sample collection.

Institutional Review Board Statement: Not applicable. **Informed Consent Statement:** Not applicable.

Data Availability Statement: The data presented in this study is available on request from the corresponding author.

Conflicts of Interest: The authors declare no conflict of interest. The funders had no role in the design of the study; in the collection, analyses, or interpretation of data; in the writing of the manuscript, or in the decision to publish the results.

Sample Availability: Not available.

Supplementary references

1. Epure, L.; Meleg, I.N.; Munteanu, C.M.; Roban, R.D.; Moldovan, O.T. Bacterial and fungal diversity of Quaternary cave sediment deposits. *Geomicrobiol. J.* **2014**, *31*, 116–127.
2. Epure, L.; Munteanu, V.; Constantin, S.; Moldovan, O.T. Ecophysiological groups of bacteria from cave sediments as potential indicators of paleoclimate. *Quat. Int.* **2017**, *432*, 20–32.
3. Moldovan, O.T.; Constantin, S.; Panaiotu, C.; Roban, R.D.; Frenzel, P.; Miko, L. Fossil invertebrates records in cave sediments and paleoenvironmental assessments – a study of four cave sites from Romanian Carpathians. *Biogeosciences* 2016, *13*, 483–497
4. Sarbu, S.M.; Kinkle, B.K.; Vlasceanu, L.; Kane, T.C.; Popa, R. Microbiological characterization of a sulfide-rich groundwater ecosystem. *Geomicrobiol. J.* **1994**, *3*, 175–182.
5. Lascu, C. Paleogeographical and hydrogeological hypothesis regarding the origin of a peculiar cave fauna. *Misc. Speol. Rom.* **1989**, *1*, 13–18.

6. Soficaru, A.; Dobos, A.; Trinkaus, E. Early modern humans from the Peştera Muierii, Baia de Fier, Romania. *Proc. Nat. Acad. Sci.* **2006**, *103*, 17196–17201.
7. Constantin, S.; Mirea, I.C.; Petculescu, A.; Arghir, R.A.; Mantoiu, D.S.; Kenesz, M.; Robu, M.; Moldovan, O.T. Monitoring human impact in show caves. A study of four Romanian caves. *Sustainability* **2021**, *13*, Article number 1619.
8. Mirea, I.C.; Robu, M.; Petculescu, A.; Kenesz, M.; Faur, L.; Arghir, R.; Tecsa, V.; Timar-Gabor, A.; Roban, R.D.; Panaiotu, C.G.; Sharifi, A.; Pourmand, A.; Codrea, V.A.; Constantin S. Last deglaciation flooding events in the South Carpathians as revealed by the study of cave deposits from Muierilor Cave, Romania. *Palaeogeogr. Palaeoclim. Palaeoecol.* **2020**, *562*, Article number 110084.
9. Goran, C.; Povară, I. Mehedinti Plateau: Epuran-Topolnita karst system. In *Cave and karst systems of Romania*, 1st ed.; Ponta G.M.L., Onac B.P., Eds.; Springer International Publishing: Cham, Switzerland, 2019; pp. 183–201
10. Frentiu, T.; Pintican, B.P.; Butaciu, S.; Mihaltan, A.I.; Ponta, M.; Frentiu, M. Determination, speciation and distribution of mercury in soil in the surroundings of a former chlor-alkali plant: assessment of sequential extraction procedure and analytical technique. *Chem. Cent. J.* **2013**, *7*, Article number 178.
11. Frentiu, T.; Ponta, M.; Sarbu, C. Prediction of the fate of Hg and other contaminants in soil around a former chlor-alkali plant using Fuzzy Hierarchical Cross-Clustering approach. *Chemosphere* **2015**, *138*, 96–103.
